# Comprehensive bioinformatic analysis reveals oncogenic role of H2A.Z isoforms in cervical cancer progression

**DOI:** 10.22038/IJBMS.2021.58287.

**Published:** 2021-11

**Authors:** Eric G. Salmerón-Bárcenas, Ana E. Zacapala-Gómez, Daniela Lozano-Amado, Leonardo J. Castro-Muñoz, Marco A. Leyva-Vázquez, Joaquín Manzo-Merino, Pedro A. Ávila-López

**Affiliations:** 1 Departamento de Biomedicina Molecular, Centro de Investigación y de Estudios Avanzados del Instituto Politécnico Nacional, Av. Instituto; 2 Politécnico Nacional 2508, Col. San Pedro Zacatenco, Delegación Gustavo A. Madero, Ciudad de México; 3 Laboratorio de Biomedicina Molecular, Facultad de Ciencias Químico-Biológicas, Universidad Autónoma de Guerrero, Chilpancingo, 39090, Gro,; 4 México; 5 Division of Infectious Diseases, Stanford University School of Medicine, United States; 6 Unidad de Investigación Biomédica en Cáncer, Instituto Nacional de Cancerología, México. Av. San Fernando No. 22, Col. Sección XVI, Tlalpan,; 7 14080, Mexico City, Mexico

**Keywords:** Cervical cancer, Enhancers, H2A.Z, H2A.Z isoforms, Promoters, Proliferation

## Abstract

**Objective(s):**

Cervical cancer ranks as the fourth most common neoplasia in women worldwide in which epigenetic alterations play an important role. Several studies have reported pro-oncogenic role of the histone variant H2A.Z in different types of cancer; however, the role of H2A.Z in cervical cancer remains poorly studied. This study aimed to determine the potential role of H2A.Z in cervical cancer through a bioinformatic approach.

**Materials and Methods:**

H2A.Z expression was analyzed in The Human Protein Atlas, The Cancer Genome Atlas, and Gene Expression Omnibus datasets. The promoter regions of H2AZ1 and H2AZ2 genes were downloaded from Expasy, and the prediction of transcription factor binding motifs was performed using CONSITE, Alibaba, and ALGGEN. ChIP-seq and RNA-seq data from HeLa-S3 cells were downloaded from ENCODE. The discovery motif was investigated using MEME-ChIP. The functional annotation was examined in Enrich.

**Results:**

The expression of H2A.Z is elevated in cervical cancer. Interestingly, DNA methylation, copy number, and transcription factors AP2α and ELK1 are involved in H2A.Z overexpression. Additionally, H2A.Z is enriched on promoter and enhancer regions of genes involved in pathways associated with cancer development. In these regions, H2A.Z enables the recruitment of transcription factors such as NRF1, NFYA, and RNA Pol II. Finally, H2A.Z allows the expression of genes associated with proliferation in patients with cervical cancer.

**Conclusion:**

Our findings suggest that H2A.Z overexpression and its presence in promoters and enhancers could be regulating the transcription of genes involved in cervical carcinogenesis.

## Introduction

Cervical cancer (CC) is a public health problem, representing the fourth most common cancer among women worldwide with an incidence of 570,000 cases and 311,000 deaths reported in 2018 (1). Importantly, CC ranks second in incidence and mortality in developing countries thus representing a major public health threat (2). The 5-year survival rate is low (68.2%) mainly due to a deficient early diagnostic (3). The main risk factor for developing CC is persistent infection with high-risk Human Papillomavirus (HPV) (4). However, it is well known that infection with high-risk HPV is not sufficient for cervical carcinogenesis, since epigenetic alterations that contribute to the transformation process have been identified. Epigenetics consist of heritable changes in gene expression without modifying the DNA sequence (5). Epigenetic alterations in CC include DNA methylation, histone post-translational modifications, and non-coding RNAs (6, 7). It has been demonstrated that the histone variants also play an important role in cancer onset (8, 9). For instance, the histone variant H2A.Z is involved in transcriptional control, DNA repair, and regulation of heterochromatin (9). Therefore, alterations in its expression have been associated with oncogenic processes in several types of cancer (10-15). H2A.Z is a highly conserved variant, sharing a 60% similarity with the canonical histone H2A (16).H2A.Z has two isoforms with non-redundant functions known as H2A.Z.1 and H2A.Z.2, which are encoded by H2AZ1 and H2AZ2 genes and regulated by independent promoters (17-19).

Several studies have revealed a role for H2A.Z in regulating processes leading to cancer. In pancreatic cancer, the reduction of the H2A.Z isoforms produces deregulation in the expression of genes associated with proliferation and chemoresistance (15). Specifically, overexpression of H2A.Z.2 correlates with poor survival in patients with melanoma. In addition, BRD2 and E2F1 proteins interact with H2A.Z.2 at promoter regions to regulate genes involved in proliferation (10). On the other hand, in prostate cancer the acetylation on H2A.Z (K4, K7, and K11) allows greater chromatin accessibility, contributing to the activation of androgen receptor-associated enhancers and gene expression (12). Thus, H2A.Z is relevant to regulate different regulatory elements in cancer, allowing an oncogenic phenotype. However, the effects of H2A.Z.1 and H2A.Z.2 isoforms have not been studied in CC.

In this study, we integrated public data from different omic approaches to decipher the role of H2A.Z in CC. We showed that H2A.Z.1 and H2A.Z.2 isoforms are overexpressed in CC specimens compared with normal tissue. We also identified that both isoforms are associated with progression and nodal metastasis status. Importantly, we demonstrate that H2A.Z is enriched at promoters and enhancers in HeLa-S3 cells, allowing an increase in the expression of cancer-associated genes. Specifically, H2A.Z is associated with enrichment of transcription factors (TF), such as NRF1, NFYA, and RNA Pol II at promoter regions. In summary, our study suggests oncogenic role of H2A.Z in CC.

## Materials and Methods

***Expression analysis***

**Figure 1:**
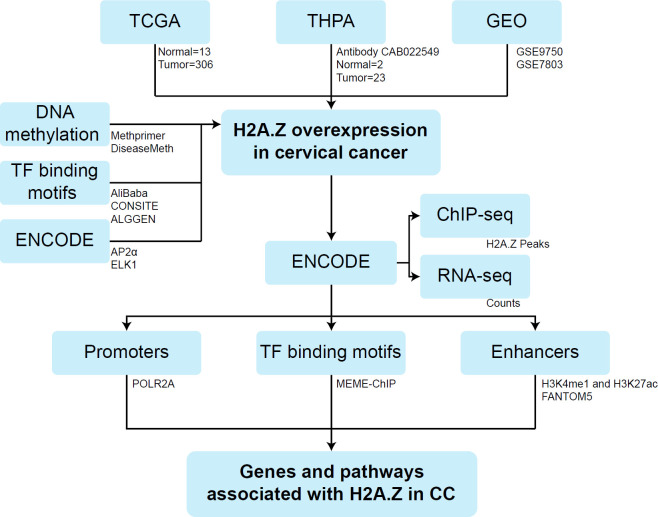
Workflow of the study

The H2A.Z expression was analyzed in CC cases using the Human Protein Atlas database (20). The specific expression of H2AZ1 and H2AZ2 was analyzed in 306 CC samples and 13 normal cervical samples using the Gene Expression Profiling Interactive Analysis (GEPIA) webserver from The Cancer Genome Atlas (TCGA) project (21). In addition, GSE9750 and GSE7803 datasets using the GEO2R software from Gene Expression Omnibus (GEO) database (22) were also analyzed (Figure 1). The GSE9750 dataset (Platform: GPL96 [HG-U133A] Affymetrix Human Genome U133A Array) includes 28 CC samples and 24 normal cervical samples (23) whilst the GSE7803 dataset (Platform: GPL96 [HG-U133A] Affymetrix Human Genome U133A Array) includes 21 CC samples and 10 normal cervical samples (24). Expression was log2 transformed and Student’s *t*-test was used to determine the differences between conditions, with a *P*-value˂0.05 considered as statistically significant. Finally, the expression of H2AZ1 and H2AZ2 was analyzed according to clinical-pathological characteristics in CC cases using the Analyze, Integrate Discover (UALCAN) database (25), and according to copy number using the cBioportal database (26-27).

***Prediction of transcription factors binding to H2A.Z promoters***

The promoter sequences of H2AZ1 and H2AZ2 were downloaded from the Expasy portal (28). A region of -2000 to +2000 base pairs was selected in relation to the transcription start sites (TSS). Then, the AliBaba2.1 (http://gene-regulation.com/pub/programs/alibaba2/index.html), CONSITE (http://consite.genereg.net/) and ALGGEN (http://alggen.lsi.upc.es/cgi-bin/promo_v3/promo/promoinit.cgi?dirDB=TF_8.3) software were used to identify potential TF binding motifs (Figure 1).

To confirm the presence of AP2α and ELK1 factors at H2AZ1 and H2AZ2 promoters in HeLa-S3 cells, the tool for interactive visual exploration of diverse genomic data, Integrative Genomics Viewer (IGV), was used (29).

***Methylation analysis***

The prediction of CpG islands at promoters of H2AZ1 and H2AZ2 was performed using Methprimer software (30). Then, methylation analysis was performed in CC cases using the DiseaseMeth2.0 database (31) (Figure 1).

***ChIP-seq data and analysis***

To determine H2A.Z enrichment on the genome of CC cells, we analyzed chromatin immunoprecipitation with massively parallel DNA sequencing (ChIP-seq) data available in ENCODE (32-33) (Figure 1). We downloaded raw data corresponding to H2A.Z ChIP-seq (accession numbers: ENCFF000BBJ and ENCFF000BBK) and control ChIP-seq (accession numbers: ENCFF000BAU and ENCFF000BAO) from HeLa-S3 cell line.

Data analysis was performed on the Galaxy platform (34). Quality control of raw data was evaluated using FastQC and then filtered for quality with Trim Galore. Afterward, the reads were mapped to the reference genome hg19 with Bowtie2 using default parameters (35). The unmapped and duplicate reads were filtered using SAMtools (36) using the following parameter: MAPQ quality score (-q) 20. The H2A.Z peaks with respect to the control were determined with MACS2 (37), with the following parameters: --nomodel, broad regions --broad, extension size --extsize 300, and peak detection based on q-value=0.05 (Supplementary Table 1). Finally, to visualize the ChIP-seq signal, deepTools2 (38) was used with the parameter --binSize 25. In order to determine the annotation genomic region of the H2A.Z peaks, we used the ChIPseeker package (39).

To determine the association between H2A.Z and the enrichment of NRF1 and NFYA in HeLa-S3 cells, we downloaded the data processed by ENCODE corresponding to bigwig of NRF1 (accession number: ENCFF000XJF) and NFYA (accession number: ENCFF000XIR). The bigwig files were viewed in IGV genome browser.

***RNA-seq data and analysis***

To determine whether the gene expression levels are associated with enrichment of H2A.Z at promoters and enhancers, we analyzed RNA sequencing (RNA-seq) data available in ENCODE (Figure 1). Raw data corresponding to RNA-seq from HeLa-S3 cells (accession numbers: ENCFF000FOM/ENCFF000FOV and ENCFF000FOK/ENCFF000FOY) were downloaded.

Data analysis was performed on the Galaxy platform. The quality control of the raw data was evaluated using FastQC and then filtered for quality with Trim Galore. Reads were mapped to the reference genome hg19 with HISAT2 using default parameters (40). Expression levels were determined with featureCounts using RefSeq as gene annotation (41) (Supplementary Table 2). Genes showing zero counts were discarded.

To determine the differentially expressed genes between CC patients with low and high H2A.Z levels we used data from TCGA-CESC (42). Briefly, we sorted the patients according to the expression levels of both H2A.Z isoforms selecting the top 20 patients with low H2AZ1, high H2AZ1, low H2AZ2, and high H2AZ2. Differential expression between “low H2A.Z.1 *vs *high H2A.Z.1” and “low H2A.Z.2 *vs *high H2A.Z.2” was performed using TCGAbiolinks and DESeq2 packages from the R program (43-44). Cut-off or differential expression was an adjusted *P*-value<0.05 and a fold change of 1.5.

***TF binding and Motif discovery***

To determine the enrichment of TFs at the promoter regions of the genes regulated by H2A.Z, we used the ChIP Enrichment Analysis (ChEA) tool (45), implemented within Enrichr (46). The detection of transcription factor binding motifs at the enrichment regions of H2A.Z was carried out with the MEME-ChIP database (47-48) (Figure 1). A 500 base pairs window from the center of the H2A.Z peaks was selected. The parameters used were motifs between 7 and 25 base pairs in width (average width 13.4) from the Human and Mouse database (HOCOMOCO v11 FULL). MEME-ChIP was searched for motifs with an E-value<0.05.

The protein-protein interactions network between H2A.Z and TFs was performed in the STRING database (49). We used the H2A.Z.1, H2A.Z.2, and TFs obtained from ChEA and MEME-ChIP as input data.

***Enhancer regions in HeLa-S3 cells***

To determine the enhancer regions in HeLa-S3 cells, we downloaded the data processed by ENCODE corresponding to bed narrowPeak of the H3K4me1 (accession numbers: ENCFF250OWR and ENCFF086NLE), H3K27ac (accession numbers: ENCFF927JDY and ENCFF101ZZI), and POLR2AphosphoS2 (accession number: ENCFF001VJB) (Figure 1).

The enrichment of H2A.Z at validated enhancers by FANTOM5 was verified (50). To visualize the signal of the H3K4me1, H3K27ac, POLR2AphosphoS2, and H2A.Z deepTools2 was used.

To determine the genes near enhancer regions we used the Genomic Regions Enrichment of Annotations Tool (GREAT) with default parameters (51).

***Functional enrichment analysis***

To determine the biological processes and pathways regulated by H2A.Z we used the Enrichr database (46). We performed an analysis of biological process and pathways enrichment considering a *P*-value<0.05 as statistically significant.

To determine the processes regulated by low H2A.Z *vs *high H2A.Z in CC patients, a Gene Set Enrichment Analysis (GSEA) (52) was performed.

***Statistical analysis***

Visualization and statistical analysis were performed in the ggplot2 package of R (53). A *P*-value<0.05 was considered statistically significant.

## Results

***The expression of both H2A.Z.1 and H2A.Z.2 isoforms is elevated in cervical cancer.***

**Figure 2:**
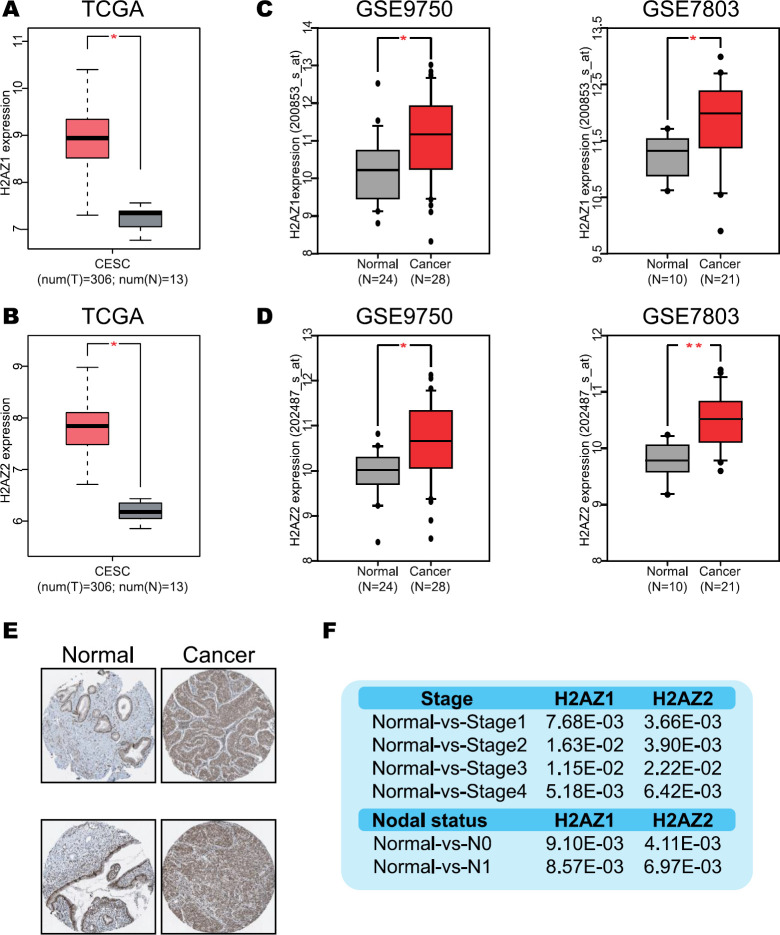
H2A.Z overexpression in cervical cancer

To decipher the potential role of H2A.Z in CC we integrated public data from different omic approaches (Figure 1). First, we evaluated the expression of both H2A.Z.1 and H2A.Z.2 isoforms in CC patients by analyzing their expression in samples from the TCGA database. Increased levels of H2A.Z.1 and H2A.Z.2 were found in CC samples compared with normal tissue (Figures 2A, B). Similar results were obtained by analyzing two microarray expression profiling datasets (GSE9750 and GSE7803) from the public GEO database, where an increase of H2A.Z.1 and H2A.Z.2 in CC samples compared with normal cervical samples was found (one-way ANOVA; H2AZ1 *P*-value˂0.05 and H2AZ2 *P*-value˂0.05, respectively; Figures 2C, D). Additionally, we identified high levels of H2A.Z in tissues from CC patients compared with normal samples in The Human Protein Atlas database evaluated by immunohistochemistry (Figure 2E). These results reveal increased levels of H2A.Z in CC, suggesting a potential role in disease progression.

To determine whether the increased levels of H2A.Z are associated with CC progression, the expression levels relative to tumor grade in the TCGA database were analyzed using UALCAN. A significant increase was found in H2A.Z.1 and H2A.Z.2 isoforms in the different stages of CC comparing with normal samples (Figure 2F and supplementary Figure 1A). Interestingly, we identified a significant increase of both isoforms according to the presence of nodal metastasis, indicating potential role of H2A.Z in tumor invasiveness (Figure 2F and supplementary Figure 1B). Together, these data suggest that the increase in H2A.Z.1 and H2A.Z.2 contributes to the CC progression.

***The increase of H2A.Z.1 and H2A.Z.2 in CC is regulated by TFs, DNA methylation, and copy number gain***

**Figure 3:**
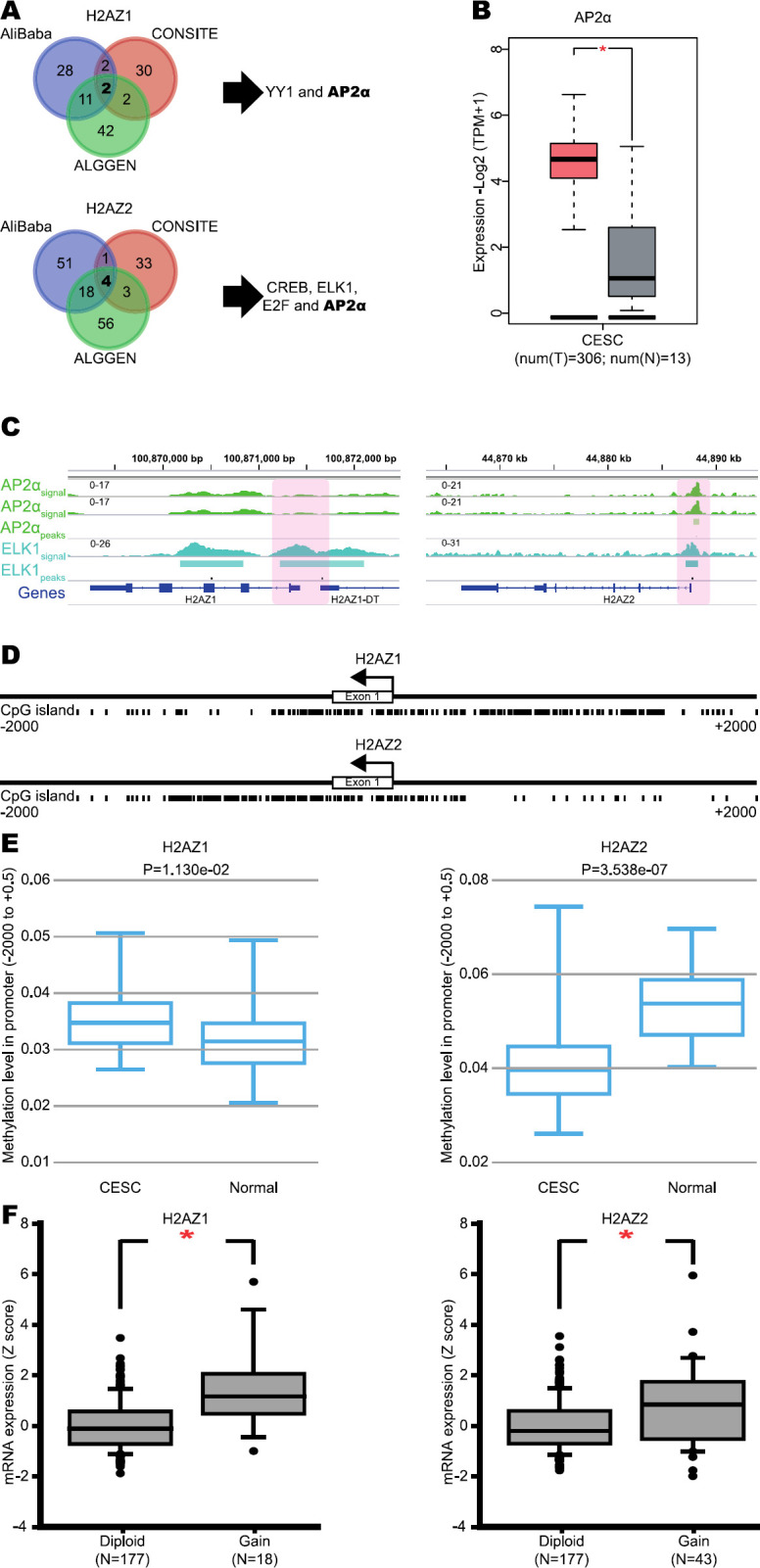
Transcription factors, DNA methylation, and copy number associate with increased H2A.Z.1 and H2A.Z.2

To evaluate potential mechanisms involved in the overexpression of H2A.Z.1 and H2A.Z.2 isoforms in CC, we first identified the TF binding motifs present at promoters of both isoforms. By analyzing the promoter regions of both genes (-2.0 to +2.0 kb relative to TSS) with AliBaba2.1, CONSITE, and ALGGEN programs, binding motifs for YY1 and AP2α were identified at H2AZ1 promoter, and binding motifs for CREB, ELK1, E2F, and AP2α at H2AZ2 promoter (Figure 3A). In addition, we evaluated the expression levels of AP2α, which was found overexpressed in CC compared with normal tissue (Figure 3B), suggesting that this TF could induce the overexpression of both H2A.Z isoforms. Interestingly, a significant enrichment of AP2α at H2AZ2 promoter in the CC cell line, HeLa-S3, was identified (Figure 3C). Moreover, a significant enrichment of ELK1 at H2AZ1 and H2AZ2 promoters was also found (Figure 3C), suggesting that AP2α and ELK1 could be regulating the overexpression of H2AZ1 and H2AZ2 in CC.

Notably, H2AZ1 and H2AZ2 promoters harbor CpG islands (Figure 3D), suggesting a possible role of DNA methylation in H2A.Z regulation. To evaluate the methylation status of H2AZ1 and H2AZ2 promoters in CC, we used the DiseaseMeth2.0 database finding a significant reduction in H2AZ2 promoter methylation in CC compared with normal tissue (Student’s *t*-test; H2AZ2 *P*-value=3.538e-07; Figure 3E right), suggesting that H2AZ2 hypomethylation could be associated with overexpression of the H2A.Z.2 isoform. Regarding the H2AZ1 gene, a significant increase was identified in the methylation grade of its promoter in CC compared with normal tissue (Student’s *t*-test; H2AZ1 *P*-value=1.130e-02; Figure 3E left), indicating that methylation status could not affect the high expression of this isoform. Finally, using the cBioportal database, we identified a significant increase in copy number gain in patients with elevated levels of H2A.Z.1 and H2A.Z.2 isoforms in CC (Student’s *t*-test; H2AZ1 *P*-value˂0.05 and H2AZ2 *P*-value˂0.05; Figure 3F). Taken together, these results suggest that overexpression of H2A.Z.1 and H2A.Z.2 isoforms could be associated with genetic and epigenetic alterations in CC.

***H2A.Z is enriched at promoters of genes associated with proliferation in HeLa-S3 cells***

**Figure 4:**
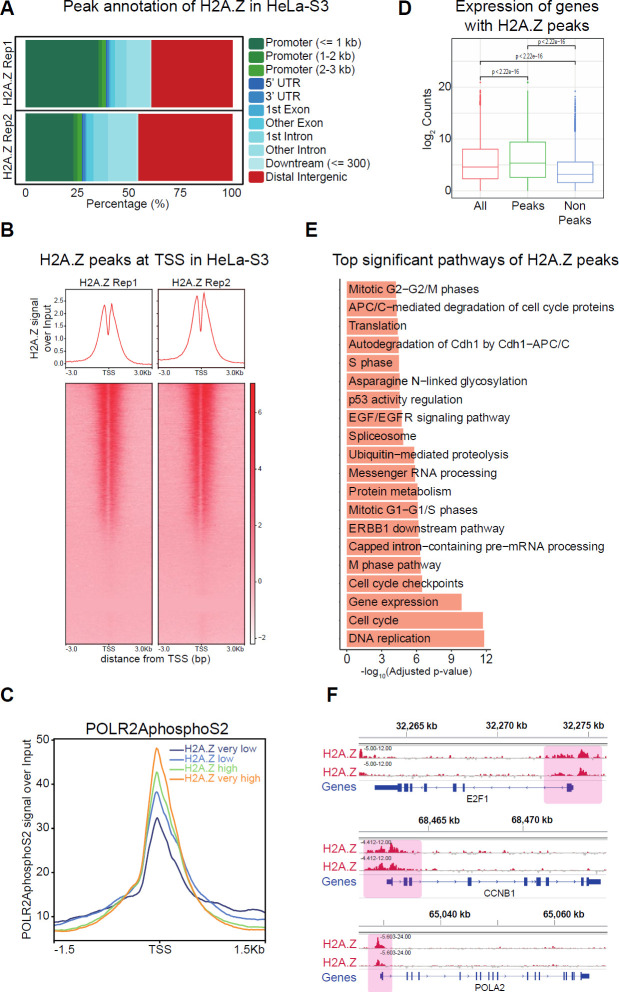
Genome occupancy of H2A.Z in HeLa-S3 cells

To understand how H2A.Z might promote CC progression, public data from H2A.Z ChIP-seq of the CC cell line HeLa-S3 were analyzed. The antibody used for ChIP-seq does not discriminate between the two H2A.Z isoforms, therefore the enrichment corresponds to both isoforms. It was identified that H2A.Z is distributed mainly at promoters (~33%) and distal intergenic regions (~42%) (Figure 4A). We found a clear enrichment of H2A.Z at promoter regions (-3.0 to +3.0 kb relative to TSS) (Figure 4B), associated with nucleosome-free regions as reported (54). Previously, it has been suggested that the enrichment of H2A.Z at promoter regions allows gene expression (10). Interestingly, we found that H2A.Z enrichment is associated with a gradual increase in the presence of POLR2AphosphoS2 at promoters (Figure 4C) (S2 phosphorylation of POLR2A predominates during transcription elongation) (55), as well as high transcription rates compared with genes that lack the presence of H2A.Z at promoters (Wilcoxon test; *P*-value<2.22e-16) (Figure 4D). Pathway analysis showed that those genes enriched with H2A.Z regulate functions associated with a proliferative phenotype such as DNA replication, cell cycle, gene expression, among others (Figure 4E). Moreover, Figure 4F shows H2A.Z enrichment at the promoters of E2F1, CCNB1, and POLA2, genes involved in the proliferation of cancer cells. Together, these results suggest that H2A.Z is associated with recruitment of RNA Pol II at promoters of highly expressed genes associated with a proliferative phenotype in CC.

***H2A.Z is associated with the recruitment of transcription factors***

**Figure 5:**
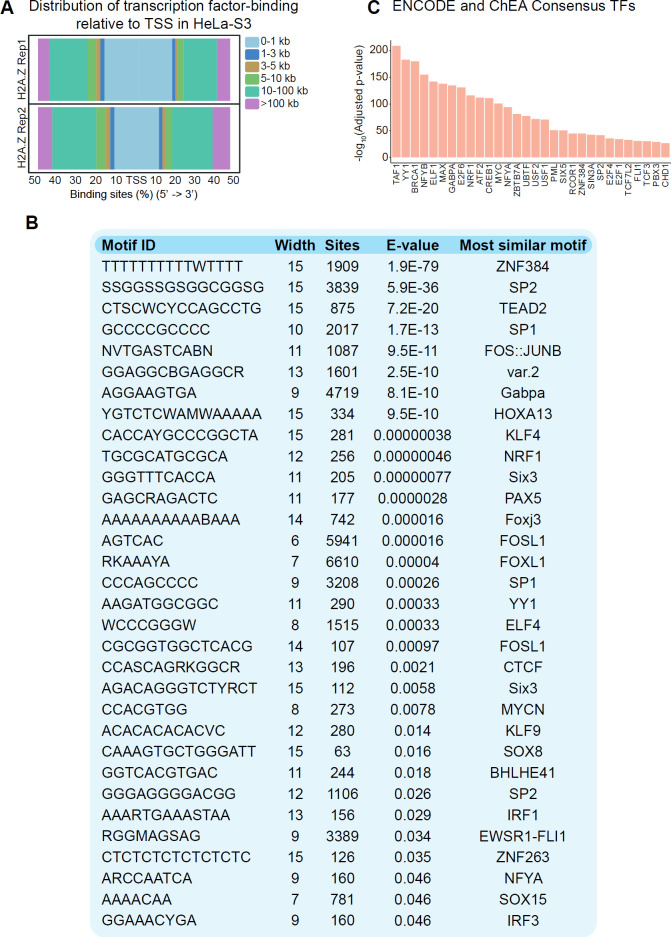
Transcription factor binding to H2A.Z peaks in HeLa-S3 cells

It has been suggested that H2A.Z can coordinate the accessibility of TFs to promoter regions (56). To determine which TFs are associated with H2A.Z peaks at promoter regions, the ChIPseeker annotation tool was used. We identified enrichment in the distribution of TF binding sites at promoters (1 kb relative to TSS) (Figure 5A). To evaluate the TF binding motifs present in these regions, we performed an analysis using MEME-ChIP. A significant enrichment of TF binding motifs, such as ZNF384, SP1/2, TEAD2, and FOS-JUNB, among others was found (Figure 5B). In addition, an ENCODE and ChEA Consensus analysis identified TFs enriched at promoters of H2A.Z-enriched genes (Figure 5C). Interestingly, these TFs establish a regulatory network with both H2A.Z isoforms (nodes: 51, edges: 206, PPI enrichment *P*-value<1.0e-16; supplementary Figure 2A), regulating important processes such as transcriptional misregulation in cancer (Benjamini-Hochberg method, adjusted P-value=3.981e-9; supplementary Figure 2B). Notably, a high H2A.Z enrichment at promoters is associated with a gradual increase in the presence of NRF1 and NFYA at these regions (supplementary Figure 2C). Overall, these data suggest that both H2A.Z.1 and H2A.Z.2 isoforms can form a complex regulatory network with TFs to regulate the expression of cancer-associated genes, thus promoting progression events in CC.

***H2A.Z is enriched at enhancers in HeLa-S3 cells***

**Figure 6:**
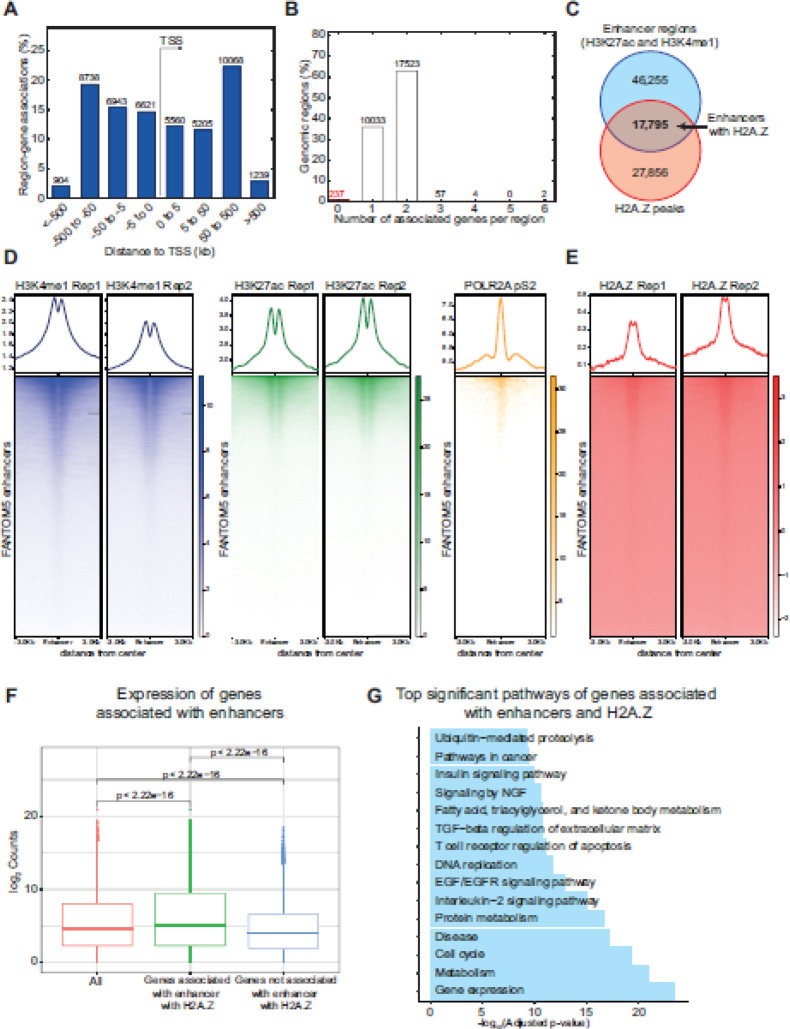
H2A.Z at enhancers in HeLa-S3 cells

Recently, it was shown that H2A.Z promotes the expression of different oncogenes by activating enhancer regions (12). To further support whether H2A.Z is located at distal regions in HeLa-S3, we use the GREAT tool identifying an H2A.Z enrichment at distal regions (Figures 4A and 6A), suggesting its presence at enhancer regions. Also, most of H2A.Z peaks are associated with regulation of 1 or 2 genes (Figure 6B), as has been described for enhancers (57). To determine the enhancer regions in HeLa-S3 cells, we analyzed enrichment peaks for the histone marks H3K4me1 and H3K27ac. We identified 46,255 enhancers (overlapping regions between both histone marks) in HeLa-S3 cells (Figure 6C). Interestingly, we identified 17,795 regions overlapping with H2A.Z peaks (Figure 6C), suggesting the presence of H2A.Z at HeLa-S3 enhancers. To confirm this result, validated enhancer regions by FANTOM5 were analyzed, which showed enrichment of H3K4me1, H3K27ac, and POLR2AphosphoS2 (Figure 6D), confirming their identity (57). As expected, H2A.Z is enriched in these validated enhancers (Figure 6E). These results suggest that H2A.Z could regulate the expression of genes associated with CC at enhancers level.

To explore whether H2A.Z promotes the expression of genes near detected enhancers, we evaluated the expression of genes associated with enhancers enriched with H2A.Z by RNA-seq, showing a significant increase in such genes (Wilcoxon test; *P*-value<2.22e-16; Figure 6F). Pathway analysis showed that genes associated with H2A.Z enhancers regulate pathways associated with carcinogenesis such as gene expression, metabolism, cell cycle, disease, among others (Figure 6G). Taken together, these results suggest that H2A.Z enrichment at enhancer regions could promote CC progression by increasing the expression of genes associated with the transformation process.

***H2A.Z.1 and H2A.Z.2 associate with genes related to proliferation in cervical cancer***

**Figure 7:**
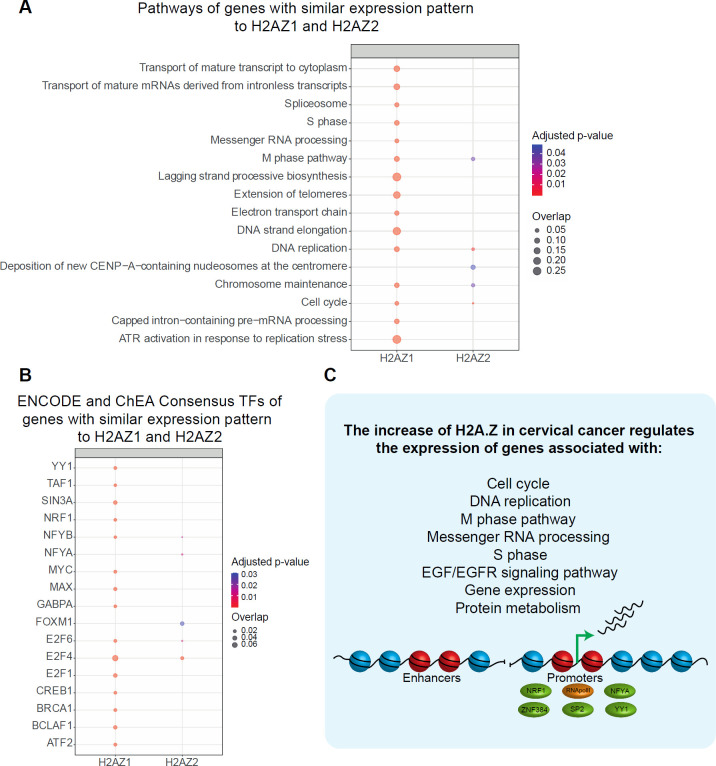
H2A.Z.1 and H2A.Z.2 associate with genes related to proliferation in cervical cancer

To investigate the biological effects associated with the increase of H2A.Z.1 and H2A.Z.2 isoforms in CC patients, we evaluated genes with similar expression patterns to both isoforms. Interestingly, we found that genes associated with both isoforms regulate common processes such as DNA replication, cell cycle, chromosome maintenance, and M phase pathway (Figure 7A), which are involved in oncogenic processes (58, 59). Furthermore, these genes are also associated with regulation exerted by oncogenic TFs, such as E2F4, E2F6, NFYB, and MYC (Figure 7B) (60). 

Additionally, we evaluated the differentially expressed genes between CC patients with low expression (top 20) and high expression (top 20) of both H2A.Z isoforms using TCGA data. We identified 765 genes associated with H2A.Z.1 overexpression (high H2A.Z.1) and 1,518 genes associated with low levels (low H2A.Z.1) in CC (adjusted *P*-value<0.05; FC of 1.5; supplementary Figure 3A and supplementary Table 3). In addition, we identified 1,446 genes associated with high H2A.Z.2 and 1,552 genes associated with low H2A.Z.2 in CC (adjusted *P*-value<0.05; FC of 1.5; supplementary Figure 3A and supplementary Table 3). GSEA analysis showed enrichment of processes associated with E2F targets, MYC targets, and DNA repair in high H2A.Z.1 and high H2A.Z.2 groups (NOM *P*-value<0.05; supplementary Figures 3B, C).

In summary, these results suggest oncogenic role of H2A.Z.1 and H2A.Z.2 isoforms in CC (Figure 7C), mainly, associated with transcriptional regulation of genes related to oncogenic processes exerted by H2A.Z at the level of promoters and enhancers (Figure 7C).

## Discussion

CC is a global health problem for which new diagnostic and therapeutic tools are needed to improve the quality of life of patients. For these reasons, epigenomic approaches may be required to understand the molecular mechanisms involved in cancer progression, which finally allows the identification of potential clinical tools (61).

Although the presence of HR-HPV infection is considered a requisite for CC development, several reports have shown that epigenetic alterations facilitate the carcinogenic process through transcriptional regulation of cancer-associated genes (61). In CC, alterations in the mechanisms of DNA methylation, histone post-translational modification, and non-coding RNAs have been reported (5), suggesting that different epigenetic mechanisms may be associated with cervical carcinogenesis such as histone variants. In this study, we found overexpression of both H2A.Z.1 and H2A.Z.2 isoforms in CC, increased levels of which are associated with progression and metastasis. Importantly, we show that H2A.Z is enriched at promoter and enhancer regions, associated with gene expression in HeLa-S3 cells. Nonetheless, association of HPV and regulation of H2A.Z remain to be clarified in CC, since there are no current data demonstrating such a relationship.

When analyzing expression data from CC patients, we identified an increase in the expression of H2A.Z.1 and H2A.Z.2 isoforms, which was associated with the stages of progression and nodal metastasis. These data agree with those reported by Yang *et al.*, where H2A.Z overexpression was associated with tumor stage, lymph node and metastasis in intrahepatic cholangiocarcinoma (13). Furthermore, Svotelis *et al.* also identified a correlation between high levels of H2A.Z and high-grade breast cancer (11), therefore our results suggest oncogenic role of H2A.Z in CC.

Few studies have addressed the molecular mechanisms responsible for H2A.Z overexpression. We identified an enrichment of AP2α and ELK1 at H2AZ1 and H2AZ2 promoters in HeLa-S3 cells that might facilitate their overexpression. Previous studies have demonstrated the oncogenic role exerted by AP2α and ELK1 in colorectal and bladder cancer (62-64). Specifically, AP2α and ELK1 form a regulatory network that facilitates the SIRPα expression in tumor-associated macrophages, which was associated with poor survival in colorectal cancer (64), suggesting that AP2α and ELK1 could promote the transcription of both H2A.Z isoforms. To date, the role of AP2α and ELK1 on gene expression in CC has not been demonstrated, thus its exploration in CC is required. 

DNA methylation has been extensively studied in CC (65-66). We identified hypomethylation at the H2AZ2 promoter. Hypomethylation often occurs at promoter regions, allowing an increase in gene expression (66). On the other hand, we identified a copy number gain of H2AZ1 and H2AZ2 genes in CC. It is well known that an increment in copy number is associated with carcinogenesis (67), thus H2AZ gain can partially explain high transcription levels. Likewise, Vardabasso *et al.* identified an increase in the copy number of H2AZ1 and H2AZ2 in melanoma patients, which was associated with the overexpression of both isoforms (10). Together, our data suggest that the increase in H2A.Z.1 and H2A.Z.2 isoforms can be regulated by mechanisms associated with TFs, as well as genetic and epigenetic alterations in CC.

Interestingly, we identified H2A.Z enrichment at promoters and enhancers of the CC cell line HeLa-S3. Our data show evidence to suggest that H2A.Z facilitates the recruitment of RNA Pol II at promoter and enhancer regions, inducing their activation. Specifically, at promoters, H2A.Z is associated with high RNA Pol II enrichment and expression of genes associated with the proliferative phenotype in HeLa-S3 cells. It has been previously reported that the presence of H2A.Z in promoters allows the expression of proliferation-related genes in bladder cancer (14). Specifically, H2A.Z is associated with high levels of H3K4me2/3 around the TSS. In addition, it coincides with recruitment of WDR5 and BPTF favoring gene expression (14). This suggests that H2A.Z promotes TF recruitment to promoters in HeLa-S3 cells.

Several studies have shown that H2A.Z allows TFs recruitment (56). Here we demonstrate that enrichment of H2A.Z at promoter regions is associated with the presence of TFs involved with transcriptional misregulation in cancer. Interestingly, a high H2A.Z enrichment is associated with high levels of NFYA and NRF1 at promoters which in turn have been associated with transcriptional alterations in CC (68-70). NFYA a trimetric transcription factor has a dual role as an activator and a repressor of transcription (71). It has been demonstrated that NFYA allows the expression of SOX2 in CC stem cells, being an important molecule for the maintenance of these cells (68). The regulatory network between H2A.Z and TFs suggested possible hub TFs and critical pathways for cervical carcinogenesis. However, an experimental approach is needed to verify the cooperation between H2A.Z and TFs such as SP2, ZNF384, YY1, NRF1, and NFYA in the transcriptional misregulation in cancer.

On the other hand, we identified that H2A.Z is associated with high expression of genes near enhancers in HeLa-S3, allowing the activation of pathways such as gene expression, metabolism, cell cycle, disease, etc. Previous studies have shown that the presence of H2A.Z at enhancers is associated with chromatin accessibility, DNA hypomethylation, RNA Pol II recruitment, and RAD21-dependent enhancer RNA transcription (72). In prostate cancer, the incorporation of H2A.Z is a requisite for activation of androgen receptor-associated enhancers. This enrichment allows the formation of the nucleosome-free region and the transcription of enhancer RNAs (12). Taken together, these results demonstrate that H2A.Z has a pro-oncogenic role in CC by regulating transcription involving promoters and enhancers.

With these results, we can propose an oncogenic mechanism associated with H2A.Z overexpression in CC, supported by H2A.Z binding to DNA regulatory elements, which in turn promotes a pro-oncogenic transcriptome, allowing the activation of genes associated with cell cycle, DNA replication, and gene expression in CC patients. Therefore, we can consider H2A.Z a potential therapeutic target for CC. For instance, in pancreatic cancer and intrahepatic cholangiocarcinoma, reduction of H2A.Z sensitizes cancer cells to chemotherapy (13, 15). Specifically, in pancreatic cancer, the reduction of H2A.Z isoforms promotes sensitivity to gemcitabine chemotherapy as well as reduction of tumorigenic processess (15). Hence, it is necessary to investigate the role of H2A.Z in proliferation, DNA replication, migration, and invasion through loss-function assays in CC cell models to further support the oncogenic role of this histone variant.

Bioinformatics workflow for deciphering the role of H2A.Z in CC

TCGA: The Cancer Genome Atlas; THPA: The Human Protein Atlas; GEO: Gene Expression Omnibus; TFBM: Transcription Factor Binding Motifs

(A) H2AZ1 expression in CC samples and normal samples from TCGA dataset. One-way ANOVA, *P*-value˂0.05. (B) H2AZ2 expression in CC samples and normal samples from TCGA dataset. One-way ANOVA, P-value˂0.05. (C) H2AZ1 expression in CC samples and normal samples from GSE9750 and GSE7803 datasets. Student's t-test, *P*-value˂0.05. (D) H2AZ2 expression in CC samples and normal samples from GSE9750 and GSE7803 datasets. Student's t-test, *P*-value˂0.05-left and ˂0.01-right. (E) H2A.Z.1 y H2A.Z.2 expression in CC samples and normal samples from The Human Protein Atlas database. Two representative images are depicted. (F) H2AZ1 and H2AZ2 expression in CC samples according to FIGO stages and metastasis status from TCGA database. Student's t-test, *P*-value is shown for each group compared with normal samples

(A) Identification of TF binding sites at H2AZ1 and H2AZ2 promoters. (B) AP2α expression in CC samples and normal samples from the TCGA database. One-way ANOVA, *P*-value˂0.05. (C) IGV tracks of the AP2α and ELK1 at H2AZ1 and H2AZ2 promoters in HeLa-S3. The red area defines the promoter region of both genes. (D) Identification of CpG islands at H2AZ1 and H2AZ2 promoters. (E) Methylation level at H2AZ1 and H2AZ2 promoters. Student's t-test, H2AZ1 P-value=1.130e-02 and H2AZ2 *P*-value=3.538e-07. (F) Expression of H2A.Z.1 and H2A.Z.2 isoforms in CC samples with copy number gain compared with diploid CC samples. Student's t-test, H2AZ1 *P-value˂0.05

(A) Genomic region annotation of the H2A.Z peaks detected in the two replicates in HeLa-S3. (B) Heatmap and profile plots of H2A.Z enrichment at promoters. The signal intensity of H2A.Z is shown with respect to the input. (C) Profile plots of POLR2AphosphoS2 enrichment at promoters. The signal intensity of POLR2AphosphoS2 is shown with respect to the input in four groups of genes sorted with respect to the enrichment of H2A.Z. (D) Box plot of the expression levels of genes with H2A.Z enrichment and not H2A.Z-bound at promoter region in HeLa-S3. Genes with counts of zero were discarded. Wilcoxon test, *P*-value <2.22e-16. (E) Functional annotation of genes with H2A.Z enrichment at promoters. The top 20 of the most significant pathways are shown. We considered an adjusted *P*-value<0.05 as statistically significant. (F) IGV tracks of H2A.Z at the promoters of E2F1, CCNB1, and POLA2 in HeLa-S3. The red area defines the promoters

(A) Distribution of transcription factor binding relative to TSS in HeLa-S3 identified with ChIPseeker. (B) Transcription factor binding motifs at H2A.Z peaks in HeLa-S3 identified with MEME-ChIP. We considered an adjusted *E*-value<0.05 as statistically significant. (C) Bar plot of the TFs associated with genes regulated by H2A.Z identified with ChEA and ENCODE databases. The top 30 of the most significant TFs are shown. An adjusted *P*-value<0.05 was considered statistically significant

(A) Orientation and distance to TSS of the H2A.Z peaks in HeLa-S3 identified with GREAT. The gene number of different regions are shown. (B) Number of associated genes with H2A.Z peaks in HeLa-S3 identified with GREAT. The gene number of different regions are shown. (C) Venn diagram for enhancers and H2A.Z peaks in HeLa-S3. The enhancers identified with H3K27ac and H3K4me1 in HeLa-S3 are represented in blue and the H2A.Z peaks in red. (D) Heatmap and profile plots of the enrichment of H3K4me1, H3K27ac, and POLR2AphosphoS2 at FANTOM5 enhancers. The signal intensity is shown with respect to the input. (E) Heatmap and profile plots of enrichment of H2A.Z at FANTOM5 enhancers. The signal intensity of H2A.Z is shown with respect to the input. (F) Box plot of expression levels of genes associated with H2A.Z enhancers in HeLa-S3. Genes with counts of zero were discarded. Wilcoxon test, *P*-value <2.22e-16. (G) Functional annotation of genes associated with H2A.Z enhancers in HeLa-S3. The top 15 of the most significant pathways are shown. We considered an adjusted *P*-value<0.05 as statistically significant

(A) Functional annotation of genes with similar expression pattern to H2A.Z.1 and H2A.Z.2 in CC. The top of the most significant pathways is shown. We considered an adjusted *P*-value<0.05 as statistically significant. (B) TFs associated with genes with similar expression patterns to H2A.Z.1 and H2A.Z.2 in CC. The top of the most significant TFs is shown. We considered an adjusted *P*-value<0.05 as statistically significant. (C) Proposed model for the role of H2A.Z in CC. H2A.Z is enriched at promoters and enhancers in HeLa-S3. H2A.Z is associated with the recruitment of RNA Pol II, NRF1, NFYA, and other TFs at promoters to allow transcription. In enhancers, H2A.Z is associated with the transcription of near genes. Finally, H2A.Z.1 and H2A.Z.2 isoforms are associated with expression of cancer-associated genes in CC

## Conclusion

Our bioinformatic analysis shows solid evidence to propose oncogenic role of H2A.Z in CC, by regulating the expression of cancer-associated genes at promoter and enhancer levels, as well as its association with TFs such as RNA Pol II, NFYA, and NRF1.

## Authors’ Contributions

EGSB and ALPA Study conception and design; EGSB and ALPA Data curation and bioinformatic analysis; EGSB, ALPA, DLA, and LJCM Writing original draft; JMM, MALV, and AEZG Writing, reviewing, and editing. EGSB, AEZG, DLA, LJCM, MALV, JMM, and ALPA Final approval of the version to be published.

## Ethics Approval and Consent to Participate

Not applicable.

## Consent for Publication

Not applicable.

## Availability of Data and Materials

The datasets used and/or analyzed during the current study are available from the corresponding author on reasonable request.

## Conflicts of Interest

The authors declare that they have no conflicts of interest.
